# A total systems approach to laboratory automation

**DOI:** 10.1155/S1463924679000602

**Published:** 1979

**Authors:** Peter B. Stockwell

**Affiliations:** 1 Laboratory of The Government Chemist Stamford Street London SE1 9NQ UK


introduced. It might even be necessary to develop a type of mobile laboratory especially designed for use in developing countries, in which all basic laboratory tests could be carried out. This need not be more complex than a simple bench supplied complete with all necessary equipment.

The experience gathered during the development of the PotLab and MonA concepts should be useful for this type of project.

### General conditions

There was general agreement that the PotLab and MonA concepts fulfilled most specifications for photometers to be used at lower levels in the primary health care of developing countries.

It was recommended that WHO should take an active part in the implementation of PotLab and MonA concepts by:

(a) supplying information on the suitability of equipment for developing countries especially to organisations concerned with such supplies, e.g. UNICEF;

(b) supporting the development of suitable analytical procedures;

(c) involving international professional organisations, e.g. International Federation of Clinical Chemistry (IFCC), International Committee for Standardization in Haematology (ICSH), and the International Union of Immunological Societies (IUIS), in the development of methods and training programmes.

(d) seeking involvement of the health care industry in both the development aspects of projects and in overcoming the problems that have so far limited availability of equipment designed specifically for developing countries.

It was recommended that WHO should take steps to produce specifications for a general clinical laboratory system, dedicated to the lower levels of primary health care in developing countries, because of the expected importance such a system would have for the screening and treatment of common disorders.

All participants agreed that the meeting had been a valuable forum for the exchange of ideas between interested parties and similar meetings should be organised on a continuing basis.

ACKNOWLEDGMENTS

During the development of this project, many people world-wide, too numerous to mention, have helped with advice, hospitality, evaluation work, etc. Their collaboration is gratefully acknowledged.

REFERENCES

[1] Clinical chemistry for developing countries. Report of the joint International Federation of Clinical Chemistry and World Health Organization Meeting, September 20-24 1971, Geneva, Switzerland.

[2] Mitchell, F.L. The indigenisation of pathology. *Annals of Clinical Biochemistry,* 1975, **12**, 45.

[3] Mitchell, F.L. Simplified instrumentation for the clinical laboratory. *Bulletin of Pan American Health Organisation,* 1976, **10**, 212.

[4] Bull, G.M., Mitchell, F.L. The provision of clinical laboratory services in developing countries. *Tropical Doctor,* 1978, 8, 104.

[5] Brown, S.S. A British approach to technology for rural care: 'appropriate' clinical laboratory systems. CS10 Communications (1979), in press.

[6] Rideout, J.M. *The Journal of Automatic Chemistry,* 1979, 1,(4), 223.

[7] WHO Consultation on Standardization in Haematology, Bilthoven, April/May, 1975. Document LAB/75.3.

[8] WHO Consultation on Standardization in Clinical Chemistry, Geneva, February 1975. Document LAB/75.2.

[9] Pocock, S.N., Rideout, J.M. *The Journal of Automatic Chemistry*, 1979, 1(4) 222.

[10] Hjelm, M, The use of 'push the button' analysers in clinical chemistry. Organisation des Laboratoires-Biologie Prospective. III$^{e}$ Collogue de Pont-a-Mousson, 1975. L'expansion Scientifique Francaise, p.11.

[11] Wilding, P. and Kennedy, J.H. Manual of Routine Methods in Clinical Chemistry for use in intermediate laboratories. 1978, WHO LAB/78.1.

### A total systems approach to laboratory automation

**Peter B. Stockwell**

*Laboratory of The Government Chemist, Stamford Street, London SE1 9NQ, U.K.*

The concept of laboratory automation is a subject of much confusion. One of the major reasons for this is that there is no common understanding of the terminology itself as a subject except in a clinical sense. For example in the latest edition of Kirk-Othmer: Encyclopedia of Chemical Technology [1] no direct reference to automated analysis is made; it includes only a reference to biomedical automatic analysers, thus neglecting a wide area of the use of automated analyses. The International Union of Pure and Applied Chemical (IUPAC) definition of automation clearly excludes most systems commonly marketed as automatic. It states "automation is the use of combinations of mechanical devices to replace, refine, extend or supplement human effort and facilities in the performance of a given process, in which at least one major operation is controlled, without human intervention, by a feedback system". Almost all developments described as automatic are classified within the IUPAC definition of mechanisation; this states "mechanisation is the use of mechanical devices to replace, refine, extend or supplement human effort". A more simple definition of automation which is widely accepted by workers in the field is, "the use of any facility either electronic or mechanical, which eliminates some aspect of manual interaction and improves the efficiency of the analytical process". In this context, feedback mechanisms have little positive advantage to offer at present, although the use of microprocessor techniques clearly modifies this situation. According to the definition which has been adopted for this paper, automation in laboratory analysis can be applied at any of the various stages throughout a procedure and attempts to minimise the level of routine operator intervention. In addition, it attempts to improve the level of quality control by the use of mechanical, electronic and computer techniques in a cost effective manner. Automatic analysis is further confused, deliberately by commercial suppliers on occasions, by the automation aspects of an instrument purporting to be fully automatic but referring to only part of the analytical procedure; most often, this is the measurement stage. A

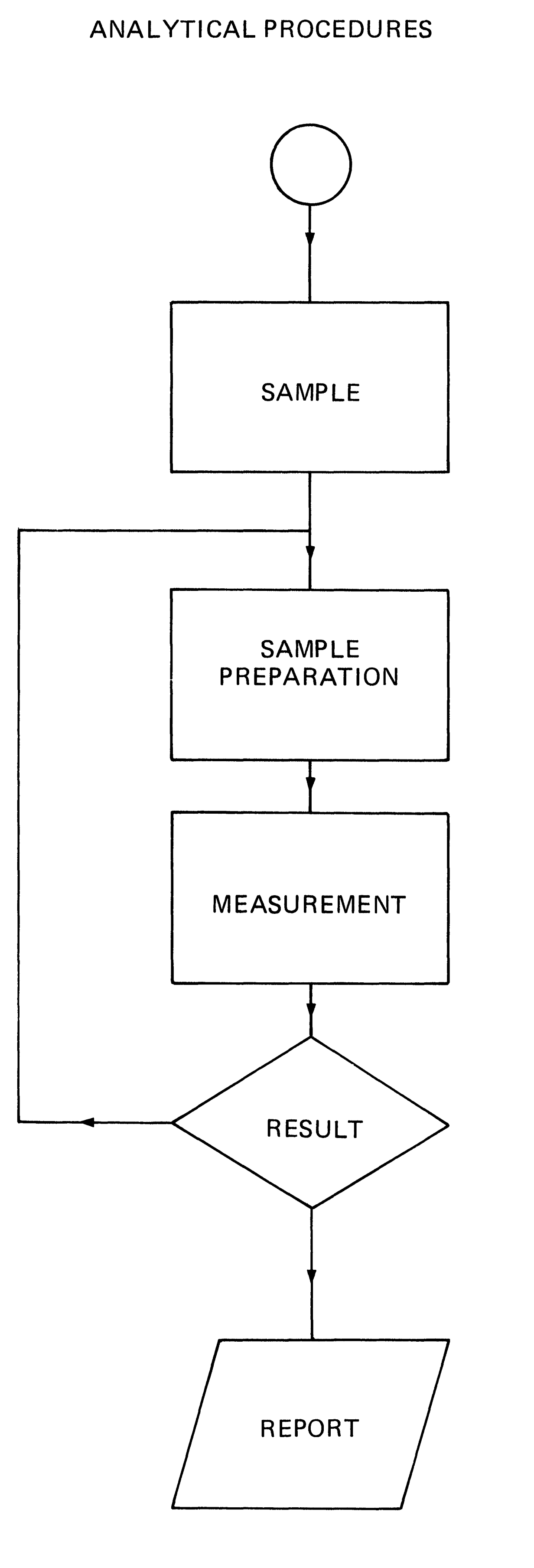

*Figure 1. Factors involved in analytical procedure.*

schematic representation of an analytical procedure as shown in Figure 1. Currie [2], defines the procedure as a systems problem and discusses the analytical process in some depth. The total systems approach to automation considers the analytical problem in its widest sense, including interaction with the customer both at the sampling and reporting stages.

In commercial chromatographic systems, the term automation generally implies electronic or computer data processing with, in some instances, the addition of injection by a mechanical syringe. Such systems provide only limited automation. The calculation of peak areas is a time-consuming activity but post-run calculations are often necessary. Injection of the sample through a syringe into a gas chromatographic column whilst being a skilled exercise occupies only a few seconds. In many situations the most time consuming activity is the sample preparation and pretreatment prior to sample injection. In addition, the calculation of peak area is only the initial stage of any calculation routine. One commercial company has recognised this situation and an automated liquid chromatographic has been described by Burns [3].

Good automation to provide the most efficient use of an instrumental system, relies on a proper and detailed specification of the analytical requirements, followed by the support of a multidisciplinary activity to meet the specification as defined. A total systems approach implies consideration of the needs of all groups involved: the needs of the customer, the analyst, the laboratory manager and the laboratory itself. For many reasons the full extent of the analytical problems is not presented by the analyst in his discussions with commercial suppliers or an in-house automation group. For example, he may approach the automation group and ask to be provided with an integrator to measure gas chromatographic peak areas, the analytical requirement eventually being to calculate the water in tar delivered from cigarettes using a gas chromatographic separation and measurement. The analyst, who may in fact have little awareness of the scope of automation or the facilities offered by available technology, presents 'how he sees the problem being solved' rather than setting out the real analytical needs. These two proposals present very different problems. Since the analytical problem may not be a single analysis and may require the combination of a number of analytical measurements, the central coordination function of an in-house computer system is important. This is exemplified by reference to the analytical and reporting procedures required for the survey of commercial cigarettes to determine the ranking of the tar and nicotine contents. The analyst is concerned primarily with single analytical results but, in addition, the laboratory manager requires the statistical mean moisture level of a brand of cigarettes as one of the analytical parameters used in determining the accepted ranking parameter, dry tar. This is obtained by subtracting the moisture and associated nicotine level from the level of tar delivered.

A total systems approach does not automatically result in a fully computerised microprocessor controlled system if this is uneconomic. In some problems the most cost effective answer is the introduction of mechanical dispensing and switching systems controlled by simple cam timer logic. A system for testing dental materials and their resistance to sudden and prolonged temperature changes is an example of this approach [4]. In other situations, as a direct result of the systems study, the analysis may remain in a manual regime but with the methodology changed significantly to improve reliability and precision.

### Specification of the problem

This is undoubtedly the most difficult part of designing and installing any automation scheme. For any new analytical requirement where automation is applied at the outset this may not be such a serious problem as where automation is

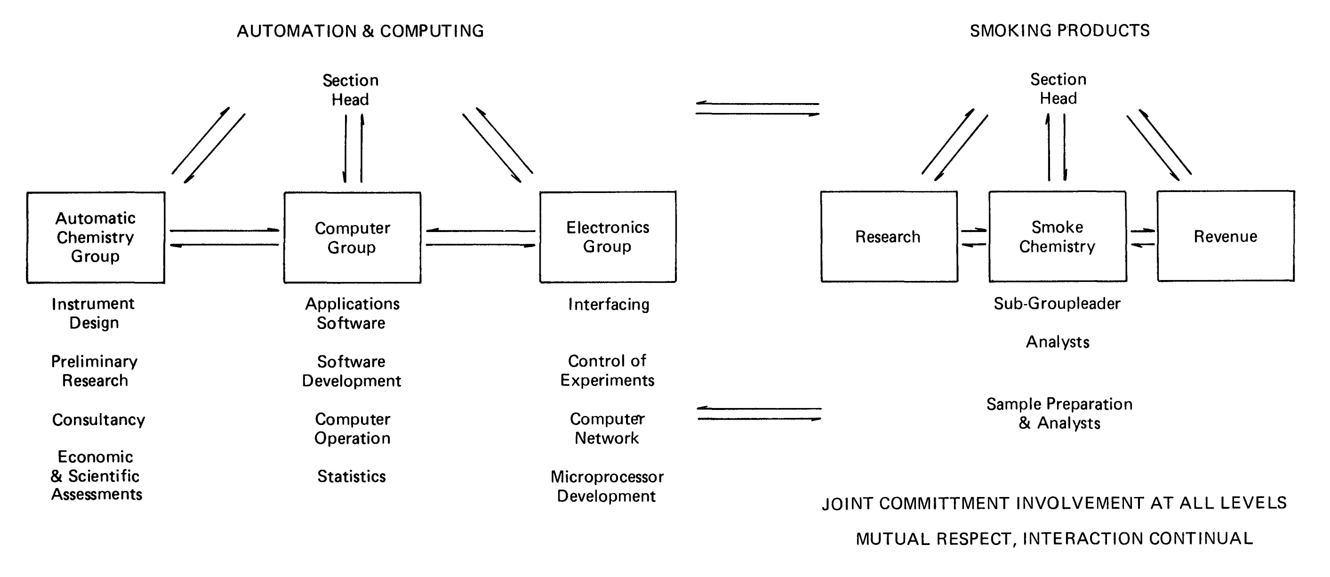

*Figure 2. Various functions and inter-relationships between automation group and user group.*

applied to procedures which have been undertaken manually for many years. In the latter cases it may be very difficult to distinguish between the analytical needs and what the analyst actually does manually. This is due to the analyst being unaware of what is possible and economic in an automated approach. There are, no doubt, many ways of meeting the objective of defining a proper specification, but the approach taken in this laboratory has been successfully applied to the automation of a range of analytical problems. Whatever approach is preferred, it is vitally important that the automation group team or systems designer, whether in-house or provided through a commercial organisation, *must* have a good understanding of analytical chemistry.

The automation and computing group at the Laboratory of the Government Chemist, was formed in 1969 with a small team of analysts, each of whom had an interest in applying automation to analytical problems. As the group has developed and expanded, the specialist skills, notably in computer programming, electronics, statistics and mechanical engineering have been incorporated into its functions. Particular care has been taken to retain the underlying expertise in analytical chemistry. In almost all projects or programmes the project leader of choice is a senior systems designer with a good analytical background. More recently, the skills associated with microelectronics and microprocessors have also been acquired. However, while the designer must resist incorporating such novel devices in automated instrumental systems where they are clearly superfluous, he must be aware of new technology and use it as a cost effective solution to the problem under consideration.

The customer, the analyst and the end user of the results (if this is not the originating customer) therefore have an extremely important role in any automation scheme. Without their full co-operation the success of the venture can be seriously impaired. In addition to automated chemical procedures and sample handling techniques, the proposed scheme of automation may very well combine electronics, computing, and statistics through a sampling and quality control programme. The skill require to develop the necessary automation thus requires the formation of a multidisciplinary team which must interface with the users. Jointly, the detailed specification must be defined and then development and implementation of the level of automation to meet this specification must proceed. Only when the groups concerned have an effective dialogue with each other, are aware of the constraints placed on each other and are totally committed to the value of automation, will the end result of the development be good automation. The resulting instrument will work reliably in a routine situation and its usefulness could be clearly demonstrated. The various skills required in the automation group are illustrated by reference to Figure 2, and have been discussed by Stockwell [5]. The approach to automating the tobacco smoke analysis in this laboratory serves to illustrate the total systems approach and its value, particularly to the staff in the tobacco section. Also shown in Figure 2 is the interaction between the two subdivisions in the work programme. A sense of involvement, even on the part of the most junior analyst and continual interaction between all members of the team must be encouraged from the specification stage through to the implementation of the automation developed.

### Automation of the survey to determine the tar and nicotine levels in cigarettes available in the UK

Publication of the report *'Smoking and Health Now'* by the Royal College of Surgeons in 1971 [6] encouraged the Department of Health and Social Security in England to commission the Laboratory of the Government Chemist to conduct a survey of all commercial cigarettes available on the UK market. The object of this survey is to publish a league table at six-monthly intervals which lists all brands according to their tar and nicotine levels. The table would also serve to advise the public of the relative health risks encountered by smoking cigarettes of different brands.

In the initial stages of the survey the level of automation was restricted. The smoking and analytical protocols originally adopted had to be similar to those in general use throughout the tobacco industry and these were basically manual procedures. With the knowledge that the survey would be a continuing requirement, the strategy adopted in the installation of an automated scheme took account of the long term advantages of automating the analytical techniques themselves as well as the sample preparation and data processing aspects. The development of the automation scheme from the initial survey, where only simple preparation and data processing and reporting were automated, through to a more sophisticated online automated system as discussed here, has been presented by Copeland and Stockwell [6]. It is not intended in this paper to cover this ground again but rather to use the example as an illustration of the various

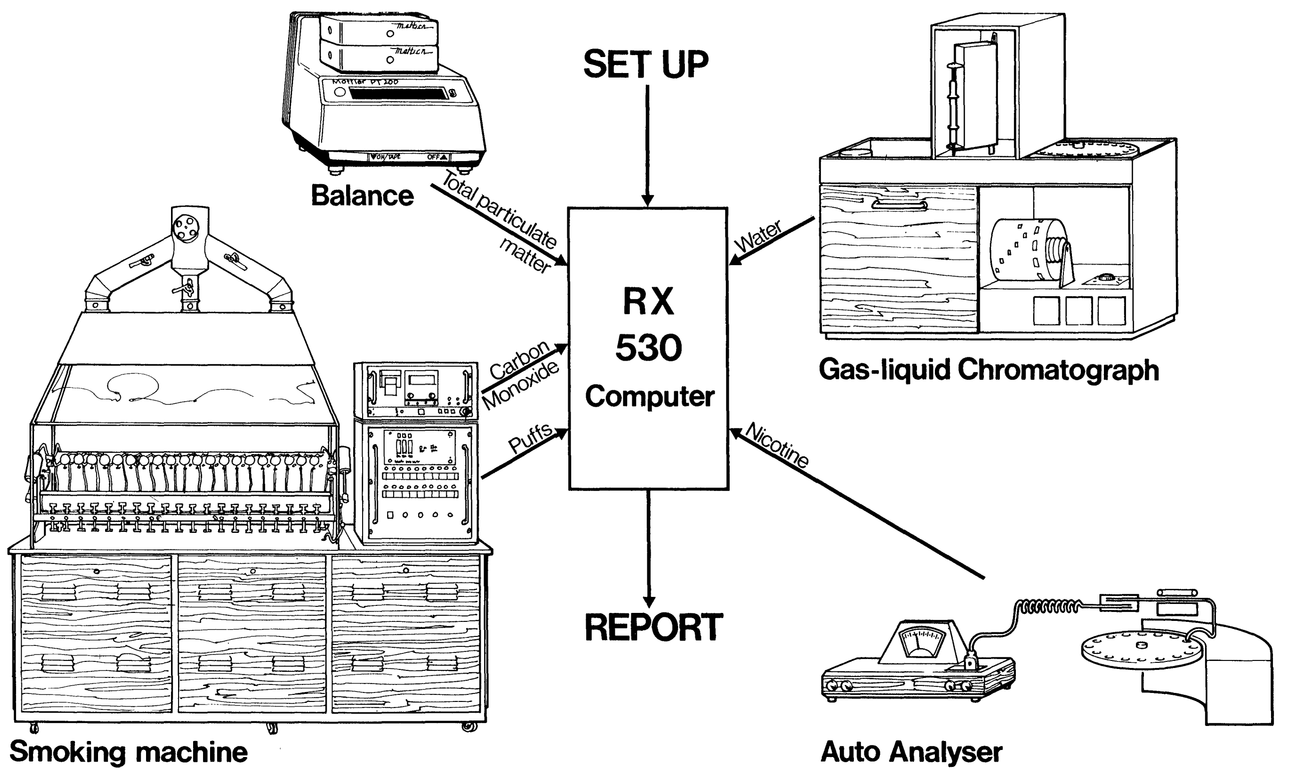

*Figure 3. Inter-relationship of automatic analyses linked online to central computer.*

aspects of the total systems approach to automation.

Because many of the automated devices have been introduced whilst the survey work was in progress, it was very important to prove their suitability for long term routine use and, most importantly, to show that the results obtained using automation were statistically similar to those obtained manually. This is important in order to confirm that no bias is introduced by the transfer from the manual to the automatic regime, examined. In practice, the relative ranking of brands must not be altered by transferring from a manual to an automatic system.

Since automation of the chemistry necessarily followed after the implementation of a sophisticated data processing and reporting structure, it is sensible that the computer should ultimately form a central co-ordinating and management function, that is act as a central registry of information. Discussions between the automation team and the user group defined a detailed specification for the requirements and economic assessments of the automation needs were carried out jointly. Where an economic case existed automation was introduced. Where appropriate and when they exist commercial instruments were installed; where not, commercial systems were modified in-house. Where commercial instruments were unavailable then automation was implemented using instruments developed entirely within the Laboratory. The solutions proposed for individual analyses have not been restricted to any one of the many philosophies of automatic analysis, and continuous flow or discrete analytical methodologies have been used as appropriate.

Table 1 illustrates the various stages involved in the experimental procedure. The first stage was to define the customer's objective as stated above. Proper experimental design ensures that the various measurements reported should be within the precision and accuracy required by the customers, the aim of these experiments being to report the mean dry tar value with a precision on that mean of less than ± 0.5 mg tar. As such, thirty measurements are made, each on five cigarettes, over the six months of the survey. Sample collection was an important facet because the values obtained for the measured analytes must be representative of the cigarettes actually available to the consumer. Collection at the site of manufacture seemed to be the best solution. Sampling instructions are issued by the centralising computer to the collecting agency and gummed labels are printed to identify and accompany the sample. These are transferred to the sample report forms. Samples of tar are generated using a conventional 20 channel automated smoking machine (Filtrona 300). This smokes each cigarette of the 5 sample set in a controlled, defined manner agreed throughout the industry. To ensure that day-to-day and inter channel variations are minimised, a pseudo random smoking programme is devised by the computer. Within each smoking programme (or run) so defined, a channel is allocated to analyse a reference cigarette, to provide a quality control check. The tar is then collected for each of the samples and retained on a filter pad prior to analysis. As a further quality control check, the puff count is measured; puff count is the total number of 35ml puffs required to smoke the cigarettes to a definite point defined as the butt mark. More recently this parameter has attained greater importance due to the requirement to measure carbon monoxide levels within the smoking procedures. This and other analytical parameters are illustrated in Figure 3 which shows all the measurements linked in an online manner to the central computer. The weight of tar produced by the cigarattes is measured directly using an online electronic balance. The dry tar value used for ranking purposes is determined from this value by subtracting the water and nicotine (defined as total alkaloids). The filter pads are extracted and an aliquot taken from water measurement using a gas chromatograph. The gas chromatograph incorporates an automated injection system which is controlled by the central computer. The computer also digitises the analogue data, integrates the peak areas and carries out subsequent data reduction and reporting. The levels of total alkaloids are measured using the continuous flow principle on a Technicon AutoAnalyzer using a cyanogen bromide procedure.

The results for each batch of 20 analyses are then presented to the analyst together with analytical calibration data. The combined analytical measurements are available to the section manager who can relate these measurements to the data base for each of the cigarette brands. The validity of each result can be compared with past data, to highlight changes in cigarette specifications and analytical variations. Graphical outputs and histograms provide a valuable additional facility available to the management. Cusum plots form an effective means of evaluating this historical data and also of comparing data with other contributing laboratories.

Once data have been validated they are incorporated into the data base for the particular brand and used in the current survey. Each monthly set of results and the accumulated statistical mean data are reported to the customer and exchanged with the industry. Finally, at the end of the survey a table, ranking the brands in ascending order of tar level (the League table), is prepared by the central computer system in a format ready for publication to the general public.

Figure 4 shows the various analytical processes and the programming format which achieves the levels of automation so far described. The process of automation has evolved since the initial survey and, because of the close liaison between the automation group and the tobacco section, it has been able to cope with the customer's changing demands. It is flexible enough to be further enhanced to meet changing needs such as the addition of further analyte measurements. Carbon monoxide has recently been included in the survey parameters; however, these are not yet published in the UK. The scope of the survey has been changed and data can now be generated for Health Tax purposes and the programmes can be extended to cover other surveys which may be undertaken on a contract basis in the future.

The experience gained in operating this analytical scheme has been extremely useful for other aspects of work in the Laboratory. Contrary to popular belief which clearly expects that automation and computerisation, in particular, require the analyst to abdicate control to a machine, the scheme devised has provided the analyst with a considerable degree of control over the procedures. It is necessary to accept the constraints on the analyst, on the customer and on the system designer, and there must be a willingness to work towards economic benefits and not exclusively towards scientific and technical interests.

Automation of the complete processes from the initial sampling including the various measurement stages through to the final reporting has been clearly demonstrated. The final product, the published league table of tar and nicotine

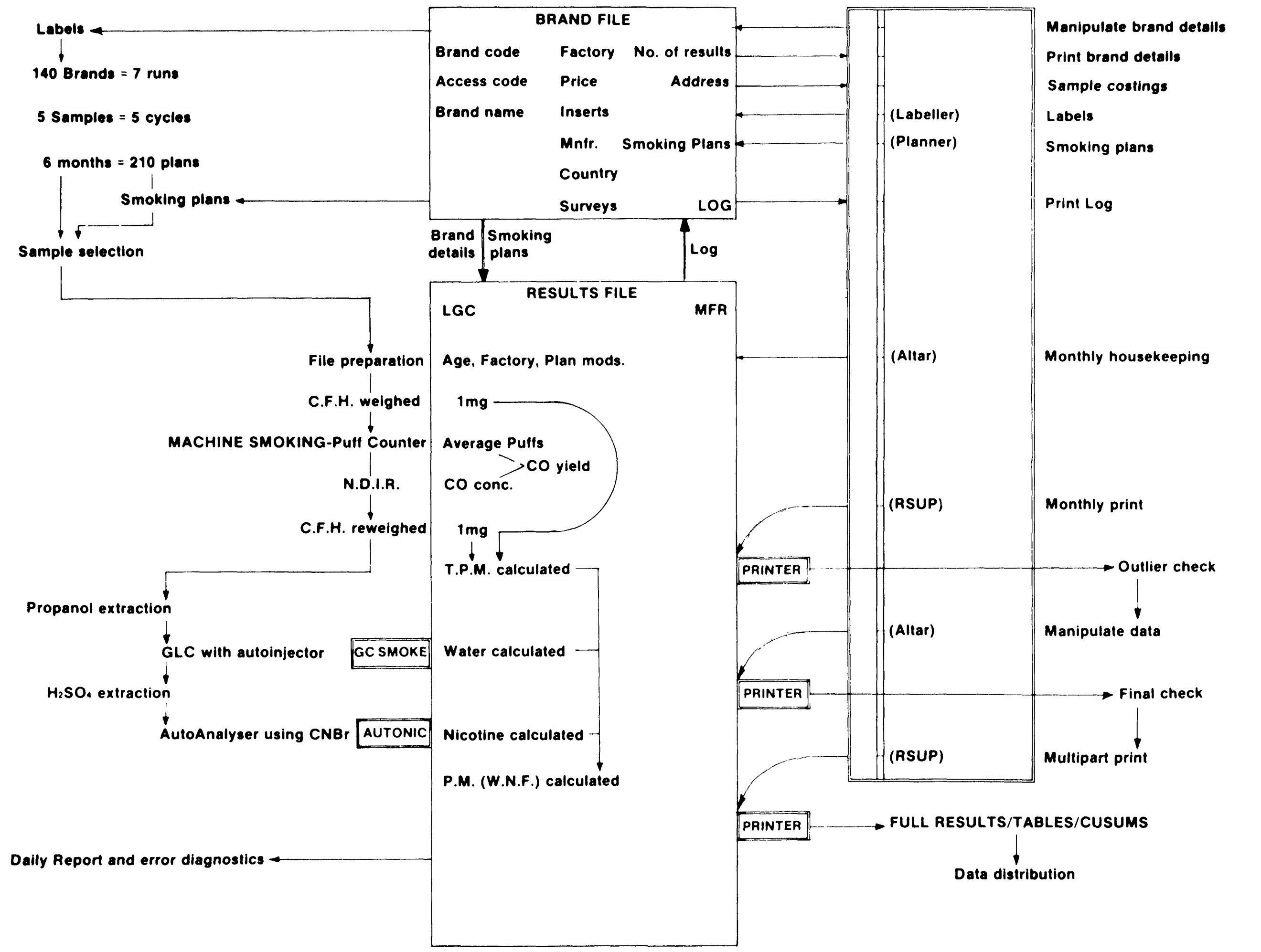

*Figure 4. Diagramatic representation of the relationship between the automatic analytical instruments, the data files, and the large interactive control programmes.*

### LOW TAR

| | | |
|---|---|---|
| Under 4 | Embassy Ultra Mild | 0.3 |
| Under 4 | John Player King Size Ultra Mild | Under 0.3 |
| Under 4 | Silk Cut Ultra Mild with Substitute | Under 0.3 |
| 8 | Embassy Premier King Size | 0.6 |
| 8 | Peer Special Extra Mild King Size (with Cytrel) | 0.6 |
| 8 | Piccadilly Mild | 0.5 |
| 8 | Silk Cut King Size with Substitute | 0.7 |
| 8 | Silk Cut No. 3 with Substitute | 0.7 |
| 9 | Belair Menthol Kings | 0.6 |
| 9 | Embassy No. 1 Extra Mild | 0.7 |
| 9 | Embassy No. 5 Extra Mild | 0.8 |
| 9 | John Player King Size Extra Mild | 0.7 |
| 9 | John Player King Size with NSM | 0 7 |
| 9 | Peter Stuyvesant Extra Mild King Size | 0.8 |
| 9 | Player's No. 6 Extra Mild | 0.7 |
| 9 | Silk Cut | 0.9 |
| 9 | Silk Cut International | 0.9 |
| 9 | Silk Cut King Size | 1.0 |
| 9 | Silk Cut No. 3 | 0.8 |
| 9 | Silk Cut No. 5 | 0.8 |
| 10 | Consulate Menthol | 0.6 |
| 10 | Consulate No. 2 | 0.6 |
| 10 | Embassy Extra Mild | 0.8 |
| 10 | Player's No. 10 with NSM | 0.7 |

### LOW TO MIDDLE TAR

| | | |
|---|---|---|
| 12 | B & H Sovereign Mild with Substitute | 1.1 |
| 12 | Gauloises Filter Mild | 0.7 |
| 12 | John Player Carlton Premium | 1.1 |
| 13 | Gauloises Longues | 0.8 |
| 13 | Gitanes International | 1.1 |
| 13 | John Player Carlton Long Size | 1.3 |
| 13 | Player's No. 10 Extra Mild | 0.8 |
| 13 | St. Moritz | 0.9 |
| 14 | Black Cat No. 9 | 1.0 |
| 14 | John Player Carlton King Size | 1.5 |
| 14 | Kent | 1.1 |
| 14 | Peter Stuyvesant King Size | 1.1 |
| 14 | Philip Morris International | 1.2 |
| 14 | Piccadilly No. 7 | 1.0 |
| 15 | Benson & Hedges Sovereign Mild | 1.4 |
| 15 | Camel Filter Tip | 1.0 |
| 15 | Chesterfield King Size Filter | 1.3 |
| 15 | Gauloises Disque Bleu | 0.8 |
| 15 | Gitanes Caporal Filter | 0.9 |
| 15 | Peer Special Mild King Size (with Cytrel) | 1.1 |

*Figure 5. End product of analyses – part of a poster illustrating the league table of the tar and nicotine levels in cigarettes available on UK market.*

Table 1. Illustration of tar and nicotine analyses problem and analytical solution.

| | |
|---|---|
| 1 Sampling | Duplicate gummed labels sent to sampling site. These serve to identify sample throughout survey and are attached to sample packet and later test note. |
| 2 Smoking programme | Computer prepares sets of randomised smoking programmes. Each of which selects the appropriate band to be smoked on each of the smoking machines twenty channels. Within each run a monitor cigarette is also smoked for quality control purposes. |
| 3 Sample preparation and analysis | Filter pads are weighed prior to smoking and again once 5 cigarettes smoked and tar collected. Analytical processes linked to central computer are shown in more detail in figure 3. |
| 4 Analytical reporting | Calibration data and individual results are available to each analyst. Laboratory manager has access to detailed brand results, is able to select data and or institute retest analyses. Trend analysis via histograms and the cusum facility also provide quality control under direction of the tobacco section manager. Files all protected by pass word entry system. |
| 5 Statistical reporting | Monthly trend results and six monthly rolling average results with summary statistics data are available. Results made available through DHSS to cooperating industry laboratories. |
| 6 Final reporting | League table ranking brands in assending order of tar produced reading for publication. |

levels is shown in Figure 5. In many laboratory situations automation to this extent may not be merited on economic grounds, however it is valuable to consider even simple analytical problems in the manner described above. Improvements in methodology rather than automation may well result by the proper definition of the analytical requirements and these in themselves may produce untold advantages. Consideration of the problems of data processing and computerisation outside the context of the complete problem, including the analytical procedure, may well result in expensive and inefficient levels of automation.

### Acknowledgements

Because of the complex nature of this automation programme and the need for such detailed and close co-operation, many people, notably all the members of the automation team, have been associated with different facets of this work. Their skills and enthusiasm for automation and computing are gratefully acknowledged.

REFERENCES

[1] Encyclopedia of Chemical Technology, Kirk – Othmer, Third Edition, John Wiley and Sons, New York.
[2] Currie, L.A., Treatise of Analytical Chemistry Part 1 Vol 1 Third Edition, Editors Kolthoff, I.M., and Elving, P.J., Wiley, New York.
[3] Burns, D.A., Seventh Technicon International Congress, New York, 1976, Proceedings publ. 1977.
[4] Morley, F., and Stockwell, P.B., *Journal of Dentistry,* 1977, 5, 39.
[5] Stockwell, P.B., *Journal of Automatic Chemistry* 1978, **1**, 10.
[6] Copeland, G.K.E., and Stockwell, P.B., Topics in Automatic Chemistry Editors Stockwell, P.B., and Foreman, J.K. publ. Horwood, Chichester, Sussex p. 265.

